# Can deep learning technology really recognize Mpox? A positive response from the EfficientNet model

**DOI:** 10.3389/fmicb.2025.1627311

**Published:** 2025-08-11

**Authors:** Xiaoqian Zhao, Long Lyu, Li Zhang

**Affiliations:** ^1^Department of Dermatology, The First Hospital of China Medical University, Shenyang, China; ^2^Key Laboratory of Immunodermatology, Ministry of Education, and National Health Commission, National Joint Engineering Research Center for Theranostics of Immunological Skin Diseases, Shen Yang, China; ^3^School of Business, Central South University, Changsha, China

**Keywords:** auxiliary diagnosis, deep learning, EfficientNet, image recognition, Mpox

## Abstract

On July 23, 2022, the World Health Organization (WHO) officially declared the Mpox outbreak a “Public Health Emergency of International Concern” (PHEIC), highlighting the urgent need for effective prevention and control measures worldwide. To assist healthcare managers and medical professionals in efficiently and accurately identifying Mpox cases from similar conditions, this study proposes a lightweight deep learning model. The model uses EfficientNet as the backbone network and employs transfer learning techniques to transfer the pre-trained EfficientNet parameters, originally trained on the ImageNet dataset, into this model. This approach allows the model to have strong generalization capabilities while controlling the number of parameters and computational complexity. Experimental results show that, compared to existing advanced methods, the proposed method not only has a lower number of parameters (only 4.14 M), but also achieves optimal values in most performance metrics, including *precision* (95.92%), *recall* (95.69%), *F1* score (95.80%), *ROC AUC* (0.998), and *PR AUC* (0.999). Furthermore, statistical analysis shows that the cross-validation results of this model have no significant differences (*p* > 0.05), which verifies the robustness of the method in Mpox identification task. Additionally, ablation experiments demonstrate that as the version of EfficientNet’s expanded network increases, the model complexity rises, with performance showing a trend of initially increasing before decreasing. In conclusion, the model proposed in this study effectively balances model’s complexity and inference accuracy. In practical applications, model selection should be based on the specific needs of decision-makers.

## 1 Introduction

On July 23, 2022, the World Health Organization (WHO) officially announced that the Mpox outbreak was upgraded to a “Public Health Emergency of International Concern” (PHEIC),^1^ which is the highest level of alert issued by the WHO (Wilder-Smith and Osman, 2020). As of August 4, more than 88 countries and regions around the world had reported Mpox infections, with the cumulative number of cases exceeding 26,000.^2^ Among the most affected countries, the United States, Spain and Germany reported over 7,100,^3^ 4,500 and 2,800 cases, respectively.

In fact, Mpox virus is not a new virus that appeared recently (Di Giulio and Eckburg, 2004; Guarner, 2022). In the earlier stages, Mpox cases were only reported in a few regions, such as the Republic of the Congo and the United States, they were only considered localized events, which did not warrant global alarm or classification as a PHEIC. As the COVID-19 pandemic continues to worsen, the emergence of a new epidemic has once again raised alarms regarding global public health security. This not only introduces additional challenges to the epidemic prevention and control efforts of governments worldwide but also places considerable strain on the frontline medical personnel tasked with managing these crises (Saxena et al., 2023; Haque et al., 2024; Kameli et al., 2025). These challenges are especially pronounced in countries or regions with fragile healthcare systems, where the experience and medical resources to address an outbreak of Mpox are insufficient.

Compared with the previous Mpox outbreaks, the current one is characterized by broader geographic spread, higher infectivity, and notable regional variations. Between late June and early July, the number of confirmed infections surged by 77%, sparking considerable concern within the international community. Moreover, according to experts, significant differences have been observed in the presentation of Mpox cases between Africa and Western countries. For example, the duration of the symptom incubation period varies. This discrepancy complicates the global response, presenting significant challenges to implementing a unified global containment strategy and complicating protective measures.

Another significant feature of the current epidemic, with considerable potential risks, is the outbreak in many countries where Mpox had never been previously reported, posing a significant challenge for governments across various regions (Wallau et al., 2022). This unusual phenomenon means that countries or regions that have never encountered the Mpox virus are now experiencing confirmed cases, creating a daunting challenge for those areas (Andrieu et al., 2025). A field survey conducted at a tertiary hospital in central China, along with random interviews with medical staff from the hospital’s dermatology department, revealed that nearly all of the medical personnel had never encountered the Mpox virus. This highlights the fact that the understanding of unfamiliar diseases among most medical staff is often limited to textbooks and other educational materials. The necessary skills and knowledge for medical and nursing work are built over time through clinical experience, meaning that medical professionals cannot readily form an effective understanding of diseases they have never seen before. As a result, the ongoing Mpox epidemic has presented substantial challenges for frontline medical staff, and misdiagnosis is a highly probable outcome.

Tedros Adhanom Ghebreyesus, the director general of WHO, has stated that some countries have limited access to diagnostic tools and vaccines, which complicates the tracking and prevention of the epidemic. This perspective underscores the critical importance and necessity of diagnostic tools (Tripathi et al., 2025). In recent years, artificial intelligence (AI) technology has rapidly developed and been applied across various fields, with most applications demonstrating the potential power of AI (Shi et al., 2025; de Vries et al., 2025; Aboulmira et al., 2025). If AI is effectively utilized in epidemic prevention and control, it could provide diagnostic support for frontline workers, not only improving their work efficiency but also reducing the risks associated with prevention and control efforts.

To address this, some researchers have attempted to use artificial intelligence techniques for Mpox identification (Sitaula and Shahi, 2022; Jaradat et al., 2023). Common models used for this task include CNN-based networks such as VGG, Xception (Mehmood et al., 2023), AlexNet (Pramanik et al., 2023), ResNet (Haque et al., 2023) series networks using residual connections to prevent overfitting, and DenseNet121 (Bala et al., 2023) that employ information-dense cross-layer connections. Although these methods have made progress by optimizing the models through increased depth, they typically require significant computational resources and time (Almars, 2025) to achieve accurate detection and classification of Mpox, which fails to meet the efficiency requirements of clinical practice.

Therefore, some researchers have focused on lightweight model structures. Maqsood et al. (2024) proposed a hybrid multi-stage fusion and selection framework using the Transformer structure to extract image features, as well as Abdelrahim et al. (2024) proposed a hybrid ensemble model based on Transformer and SVM. In fact, although Transformer models have fewer parameters, the computational complexity of their internal self-attention mechanism increases exponentially, which presents a significant challenge for small devices. To deploy Mpox identification models on edge devices, Raha et al. (2024) used the MobileNetV2 network as the backbone to extract image features. This network has fewer parameters and can achieve higher inference efficiency. Furthermore, Al-Gaashani et al. (2025) optimized and improved the model based on progressive transfer learning. Almars (2025) proposed a lightweight model combining CNN, attention mechanisms, and genetic algorithms (GA), but the GA algorithm is inherently time-consuming, which fails to truly achieve high efficiency.

Given that existing methods fail to effectively balance high efficiency and accuracy, and inspired by related research (Ravi, 2022; Wang et al., 2021; Arora et al., 2024; Kotwal et al., 2024), this study proposes a lightweight Mpox recognition model using transfer learning techniques. Specifically, on one hand, considered that the EfficientNet has achieved good results in various disease diagnoses due to its lightweight and scalability (Lee et al., 2025; Elhadidy et al., 2025; Alruwaili and Mohamed, 2025), so the EfficientNet is utilized as feature extraction module of this model. This network combines the inverted bottleneck convolution (MBConv) module with the SE attention mechanism, effectively controlling the number of parameters and computational complexity, thus achieving a lightweight model. On the other hand, to ensure high performance in small sample scenarios, transfer learning techniques are employed to transfer the pre-trained EfficientNet parameters based on the large-scale ImageNet dataset to the model. This enhances the model’s ability to capture fine-grained features and reduces the risk of model overfitting.

To evaluate the effectiveness of the proposed method, multiple experiments will be conducted. This study focuses on evaluating the performance differences of our model and its extension models in terms of *precision*, *recall*, *F1* score, *AUC*, and time complexity. Additionally, the model’s robustness is assessed using 5-fold cross-validation, and several advanced deep learning models (such as TMS and MobileNet) are chosen for comparison to validate the efficacy of the proposed model.

The structure of the remaining sections is as follows: Section 2 presents the dataset, model approaches, and process framework employed in this study. Section 3 provides a detailed analysis of the results from the baseline model and other comparison models. Section 4 concludes the paper with a summary and discussion.

## 2 Data and methods

### 2.1 Dataset introduction

Due to patient privacy concerns, obtaining a large amount of publicly available image data for Mpox virus diagnosis is challenging (Jahan et al., 2025). Fortunately, the small-scale Mpox Skin Lesion Dataset (MSLD) is openly accessible on the Kaggle platform. Kaggle is a comprehensive platform offering machine learning competitions, dataset sharing, and code collaboration, which significantly facilitates academic research and is widely recognized for its high credibility. The MSLD dataset consists of two types of images: Mpox-related and non-Mpox-related images, collected from news reports, online portals, and publicly accessible case reports across multiple media platforms. The dataset has been anonymized to protect personal privacy, eliminating the need for participant informed consent. For these reasons, this study selected the MSLD dataset as the foundation for the proposed analysis.

As previously mentioned, the MSLD dataset includes both Mpox and non-Mpox images, with the non-Mpox images primarily consisting of two diseases—chickenpox and measles—that share highly similar clinical symptoms with Mpox. In clinical practice, diseases with similar manifestations to Mpox are more prone to misdiagnosis by healthcare professionals compared to diseases with more distinct differences. Consequently, when applying artificial intelligence for the identification of Mpox, it is crucial for both researchers and models to prioritize diseases such as chickenpox and measles, which are more likely to lead to misdiagnosis by clinicians.

### 2.2 Method

**Figure 1:**
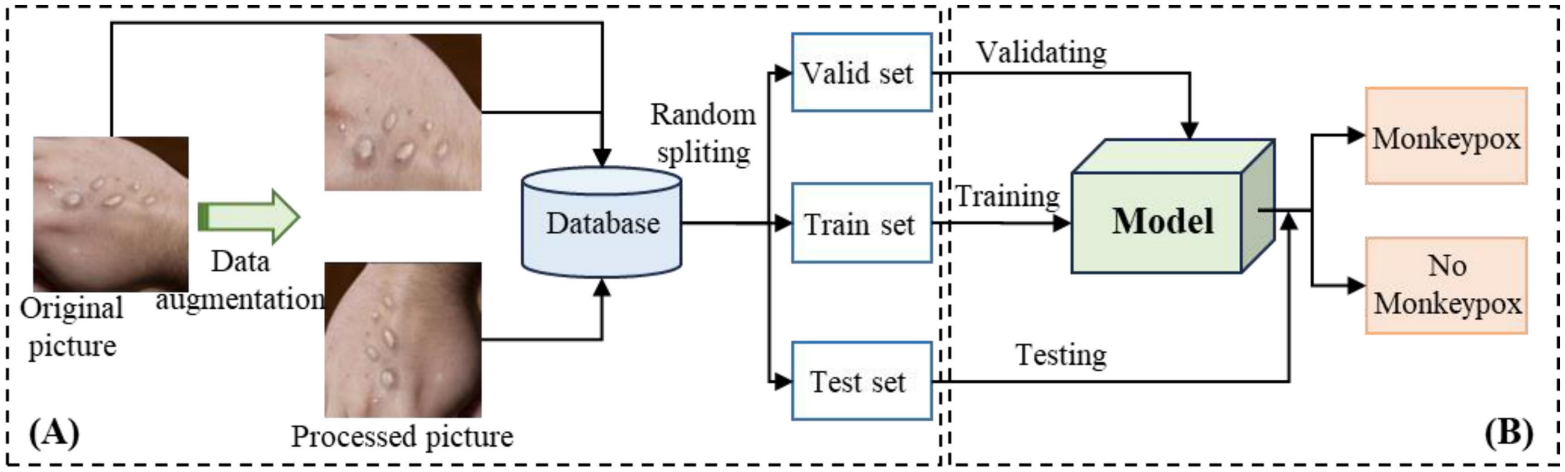
Flowchart of Mpox identification. **(A)** is the process of data preprocessing and splitting, and **(B)** is the process of modeling and validation.

The research framework of Mpox recognition is shown in Figure 1, which illustrates that the study is primarily divided into two modules: the data processing module and the training and verification module. The first module, as shown in the Figure 1A, is used for expanding the data scale. The second one, shown in the Figure 1B, is used for training and validating the model. It is worthy to emphasize that, the model’s performance is continuously evaluated throughout the training process. The second module is therefore divided into two key components: training and verification. Firstly, the training dataset is input into the model, allowing the model’s performance to gradually stabilize over multiple iterations. Secondly, to assess whether the model is overfitting during training, the validation set provides critical feedback. Specifically, if the loss exhibits a pattern of first decreasing and then increasing, it indicates potential overfitting. In such cases, further training should be stopped to prevent overfitting. Finally, the test set is used to evaluate the model’s final performance and its effectiveness in real-world scenarios.

#### 2.2.1 Data augmentation

One of the key characteristics of deep learning technology is its ability to learn the similarities and differences between various samples from large-scale datasets (Tigrini et al., 2025). When the sample size is small, it becomes challenging for the model to learn rich, deep-level features (Zhang et al., 2025). However, the MSLD dataset is relatively small, consisting of only 228 skin disease images captured in various environments, including 102 images of Mpox and 126 images of non-Mpox. Directly mining features from such a limited dataset may lead to overfitting, as the lack of feature diversity can hinder the model’s ability to generalize. As a result, data augmentation techniques are employed. These techniques serve two primary objectives: (1) to expand the scale of the dataset, and (2) to enrich the information contained within the dataset (Ribas et al., 2025; Dong et al., 2024).

Data augmentation is a method that increases data diversity without altering the intrinsic features of the original data. In the study, these methods included cropping the image into half-up, half-down, half-left, and half-right sections, as well as extracting pixel values along horizontal and vertical intervals. Other techniques applied included Gaussian blur, brightness adjustment (both brightening and darkening), flipping, adding noise, and rotating the images by 90° and 180°, among others. This technique has been successfully applied in numerous fields, especially in the field of image processing (Yang et al., 2023; Lin et al., 2025). For instance, it resulted in a 7% improvement in model performance for plant disease diagnosis (Cap et al., 2022) and a 9.46% improvement when combined with tumor segmentation studies (Zhang et al., 2022). The categorical features of the augmented images remain visually consistent with the original images, with minimal changes in classification.

After augmenting the dataset, the enhanced data is randomly divided into three subsets: the training set, validation set, and test set, with a distribution ratio of 6: 2:2. The training set is used to train the model, the validation set is employed to assess whether the model is overfitting, and the test set is used to evaluate the model’s generalization ability.

#### 2.2.2 Model

**Figure 2:**
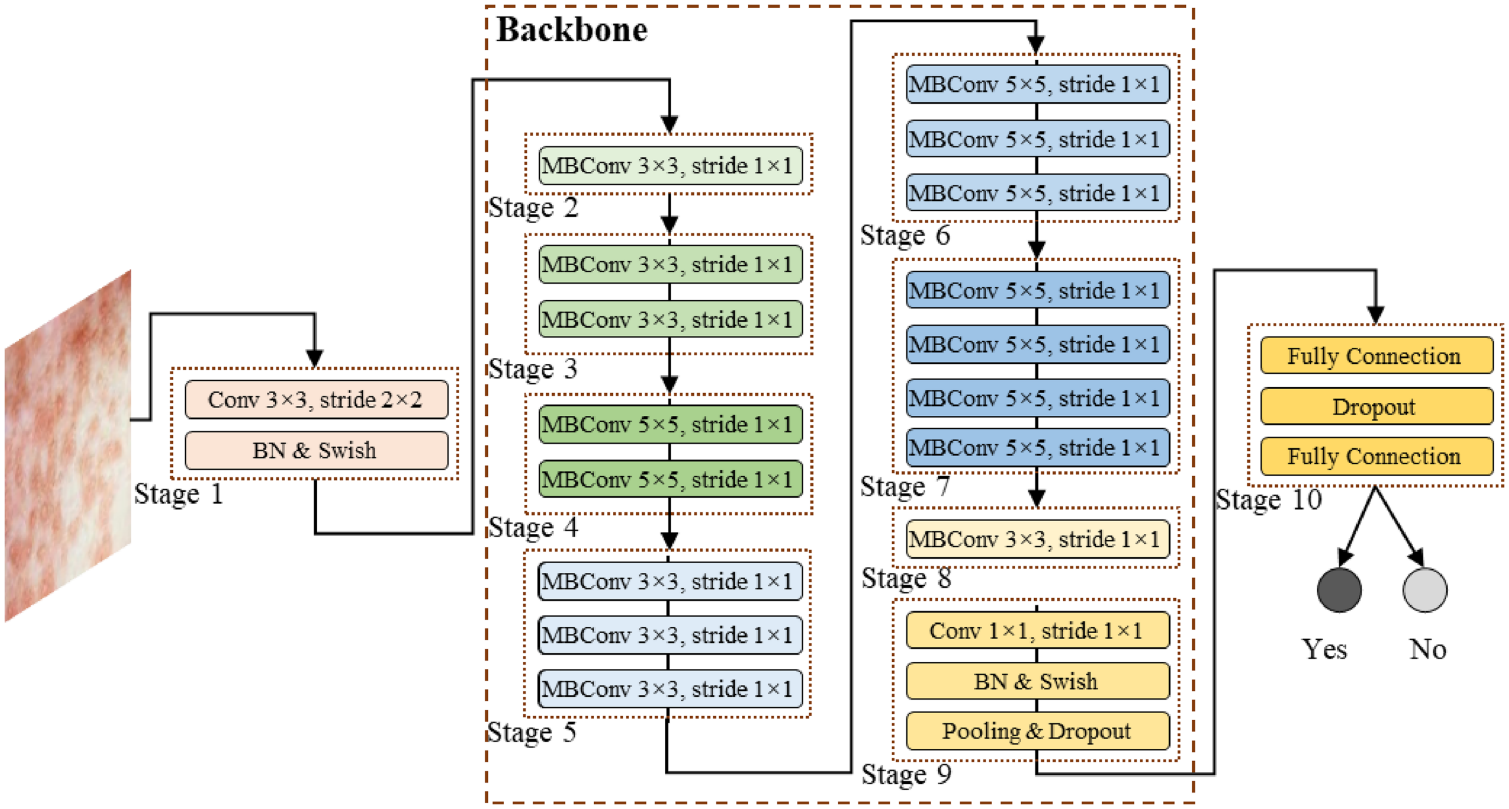
The model’s structure proposed in the study.

To achieve fast inference capabilities, high stability and accuracy, this study proposes a deep learning model for Mpox identification task based on transfer learning techniques, as shown in Figure 2. This study faces the challenge of small sample data, requiring a good balance between model performance, efficiency, and stability in the model design. The great advantages of EfficientNet in handling small sample data made the network popular in both academia and industry (Kumari et al., 2024; Wang et al., 2024; Vasavi et al., 2025). Therefore, the EfficientNet network is employed as our model’s backbone to extract local features, and the EfficientNet’s pre-trained weight parameters are transferred to our model with the utilization of transfer learning technology. It is worth noting that the pre-trained parameters of EfficientNet were obtained from the ImageNet dataset. Since this dataset focuses on larger object sizes, whereas the lesions in this study are much smaller, limited the network capability in extracting fine-grained features. Therefore, during model training, the network parameters are not directly frozen, but instead, fully involved in the model optimization process.

This model is composed of multi-layer neural network stacked sequentially, with the core component, the backbone, consisting of multiple MBConv networks. Specifically, as shown in Figure 2, the model consists of the following contents:

##### 2.2.2.1 Stage 1

As the starting stage for feature extraction, it utilizes a convolutional layer with the kernel size of 3 × 3 and the stride of 2 × 2. This layer performs initial down-sampling and feature extracting on the input image, rapidly compressing the spatial dimensions of the image while focusing on basic textures, edges, and other information. It is followed by Batch Normalization layer (BN) and the Swish activation function. BN normalizes each batch of data, accelerating model convergence and alleviating the vanishing gradient problem. Swish, with its smooth nonlinear transformation characteristics, enhances the model’s ability to express complex features, laying the foundation for subsequent deep feature extraction.

##### 2.2.2.2 Stage 2–Stage 9

**Figure 3:**
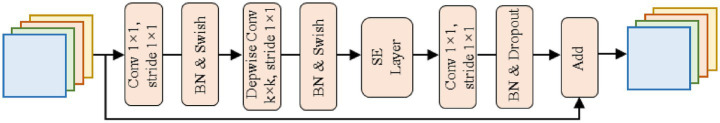
The schematic diagram of the MBConv module.

Composed of multiple MBConv modules, these stages form the core feature extraction part of the Backbone. The structure of the MBConv module is shown in Figure 3. Taking the MBConv, with the kernel size of 3 × 3 and the stride of 1 × 1, as an example, the MBConv module follows the flow with “expansion, depth convolution, attention enhancement, and projection compression.” Firstly, it expands the channel with a 1 × 1 convolution to broaden the feature learning dimensions. Then, a 3 × 3 depthwise convolution is applied to reduce computational cost while focusing on local lesion features (e.g., morphology and distribution of pustules). Thirdly, the Squeeze-Excitation (SE) attention mechanism is integrated to focus on feature channels highly correlated with Mpox diagnosis and suppress irrelevant information. The mechanism uses global average pooling layer to compress the spatial dimensions and adaptively learns the dynamic weights of different channels. Finally, a 1 × 1 convolution is used for projection compression of the channel information, outputting refined features.

The MBConv modules of different stages adjust the kernel size (e.g., Stage 4 and 6 use 5 × 5 kernels to capture broader spatial dependencies) and repetition count based on the network depth requirements, progressively extracting lesion features from shallow to deep, and from local to global.

##### 2.2.2.3 Stage 10

As the feature integration and classification output stage of the model, the high-dimensional feature vector extracted by the backbone is first flattened and mapped to the classification dimension using a Fully Connected (FC) layer, establishing the relationship between features and categories. A Dropout layer is inserted in between, randomly turning off some neurons’ information during training to effectively alleviate overfitting in small-sample scenarios and improve generalization ability. Finally, another FC layer outputs the classification prediction.

Through the collaborative operation of all layers, this model, leveraging the advantages of the EfficientNet architecture, can extract comprehensive features to achieve accurate recognition while ensuring inference speed and model stability, providing reliable technical support for Mpox diagnosis.

### 2.3 Evaluation indicators

The Mpox recognition task is a binary classification problem. In this context, evaluation metrics such as *Accuracy*, *Precision*, *Recall*, and *F1* score are commonly used to assess the performance of the model. The formulas for these metrics are presented in Equations 1–4. Specifically, *Accuracy* refers to the proportion of correctly classified cases out of the total number of cases, indicating the overall correctness of the model’s predictions. *Precision* measures the proportion of correctly identified Mpox cases among all cases predicted as Mpox. *Recall*, on the other hand, represents the proportion of true Mpox cases that were correctly identified by the model, reflecting its ability to detect Mpox. The *F1* score is a comprehensive metric that balances *precision* and *recall*, aiming to provide an overall assessment of the model’s ability to accurately classify all categories, with particular focus on identifying Mpox.


(1) $$A\text{ccuracy}=\frac{\mathrm{TP}+\mathrm{TN}}{\mathrm{TP}+\mathrm{TN}+\mathrm{FP}+\mathrm{FN}}$$

(2) $$\text{Precision}=\frac{\mathrm{TP}}{\mathrm{TP}+\mathrm{FP}}$$

(3) $$\text{Recall}=\frac{\mathrm{TP}}{\mathrm{TP}+\mathrm{FN}}$$

(4) $$F1=\frac{2\times P\text{recision}\times R\text{ecall}}{P\text{recision}+R\text{ecall}}$$

where, *TP* refers to the number of samples whose actual category is Mpox and the predicted result is Mpox. *FP* refers to the number of samples whose actual category is non-Mpox, but the predicted result is Mpox. *FN* refers to the number of samples whose actual category is Mpox and the predicted result is non-Mpox. *TN* refers to the number of samples whose actual category is non-Mpox and the predicted result is non-Mpox.

Moreover, this study incorporates the *ROC* (*Receiver Operating Characteristic*) curve and the *PR* (*Precision*-*Recall*) curve to introduce the predicted probability information. The *ROC* curve represents the relationship between the specificity and sensitivity of the model at various classification probability thresholds. The *PR* curve is constructed similarly by plotting the *precision* and *recall* at different thresholds. Both curves are derived from the predicted probabilities. The area under these curves is known as the *AUC* (*Area under the Curve*), which ranges from 0 to 1, with a higher value indicating better model performance.

## 3 Experiments and result analysis

### 3.1 Experiment setting

This study conducts all experiments using the Python programming language on a Windows system, and builds the deep learning model using the TensorFlow framework. More detailed experimental hyperparameters are shown in Table 1. The entire experiments used an RTX 3080Ti GPU with 32GB of RAM. The training set, validation set, and test set are generated using random splitting with a ratio of 6:2:2. Cross-entropy loss function and the *Adamax* optimizer are employed to optimize the model parameters, with a fixed learning rate of 0.001. To prevent overfitting, a Dropout layer is added to the final classification layer with a dropout rate of 0.4, and L1 regularization is applied to constrain the model parameters. Additionally, all network parameters, including the backbone’s parameters, are allowed to participate in the optimization process to enhance feature capturing ability in the vertical domain during training.

**Table 1:** The details of all hyperparameters related to this research.

Parameters	Details	Parameters	Details
*Backbone network*	EfficientNet	*Classifier hidden_size*	256
*Regularization method*	L1	*Batch size*	16
*Learning rate*	0.001	*Optimizer*	Adamax
*Max Epochs*	20	*Dropout rate*	0.4
*Loss*	Cross entropy	*KFold*	5
*CUDA*	RTX 3080Ti	*RAM*	32G
*Programming language*	Python	*System*	Windows

### 3.2 Data augmentation

In this study, the original image dataset was initially enhanced using a variety of techniques. The changes in the dataset size before and after processing are presented in Table 2. It is evident that the original dataset size has increased from 228 to 2,964 images, with the ratio of Mpox to non-Mpox images being 4:5. Since the data volumes of the two categories are relatively balanced, no further data balancing was required.

**Table 2:** Comparison of data scale before and after two types of image data processing.

Image category	Number of original images (sheets)	After data enhancement processing (sheet)
Mpox	102	1,326
Non-Mpox	126	1,638
Total	228	2,964

**Figure 4:**
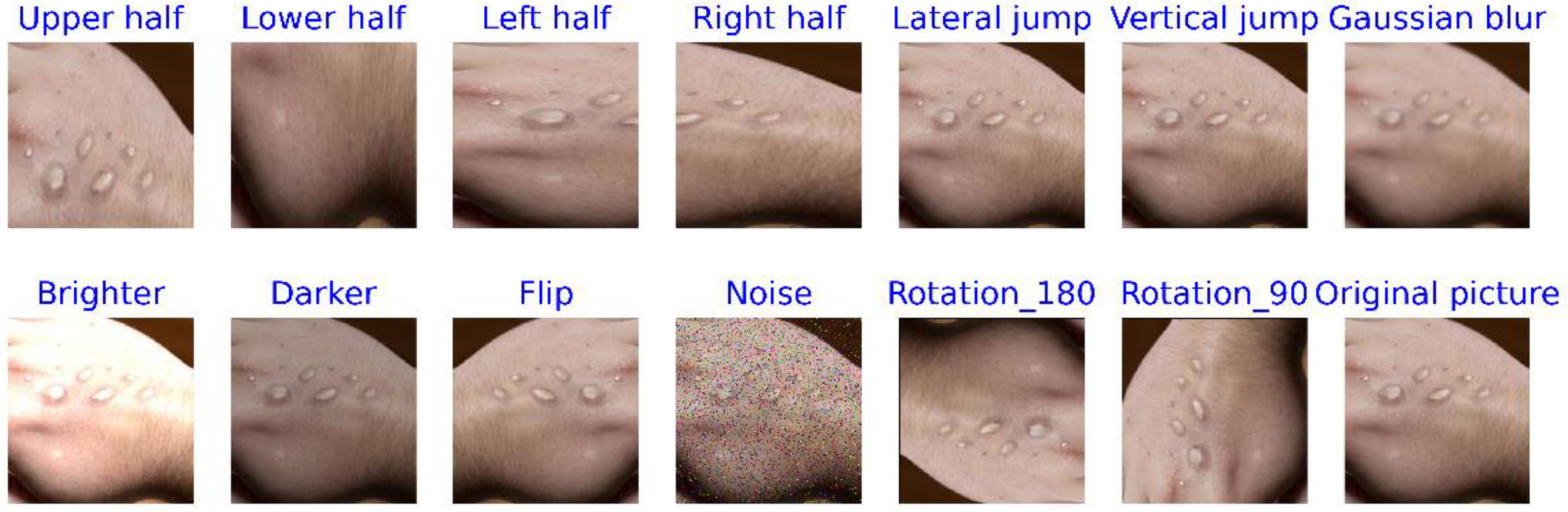
Comparison of Mpox images before and after data processing.

**Figure 5:**
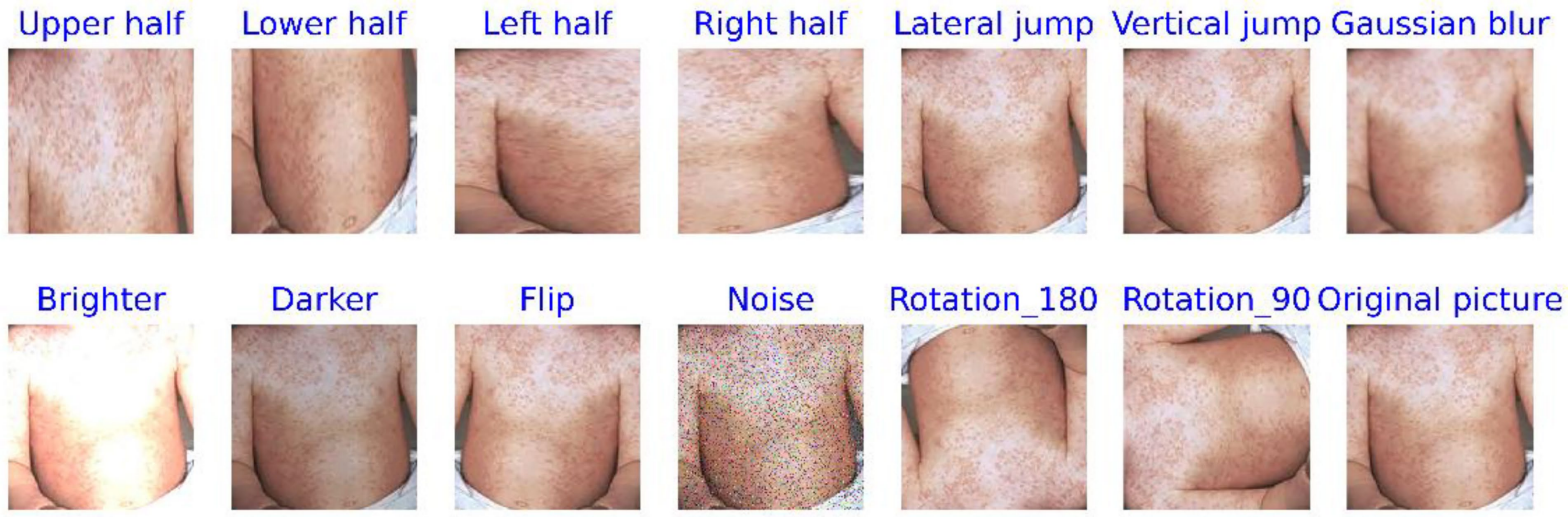
Comparison of non-Mpox images data before and after data processing.

Figures 4, 5 display comparison images of Mpox and non-Mpox data before and after data augmentation. From these figures, it can be observed that after the original images undergo data enhancement, the newly generated images, while similar to the original, are not identical. Furthermore, the categories of these augmented images remain consistent with the original, resulting in a 13-fold increase in the original dataset’s scale. This expansion provides richer feature information for model training.

### 3.3 Experiment results

#### 3.3.1 Model performance analysis

**Figure 6:**
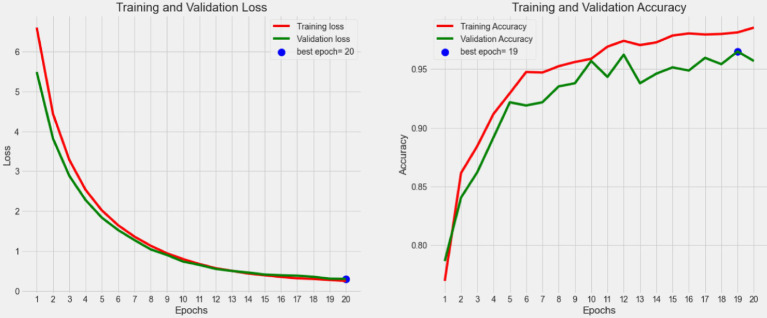
The change curve of the loss value and the accuracy rate of both the training and the validation set during the iteration of the proposed model.

**Figure 7:**
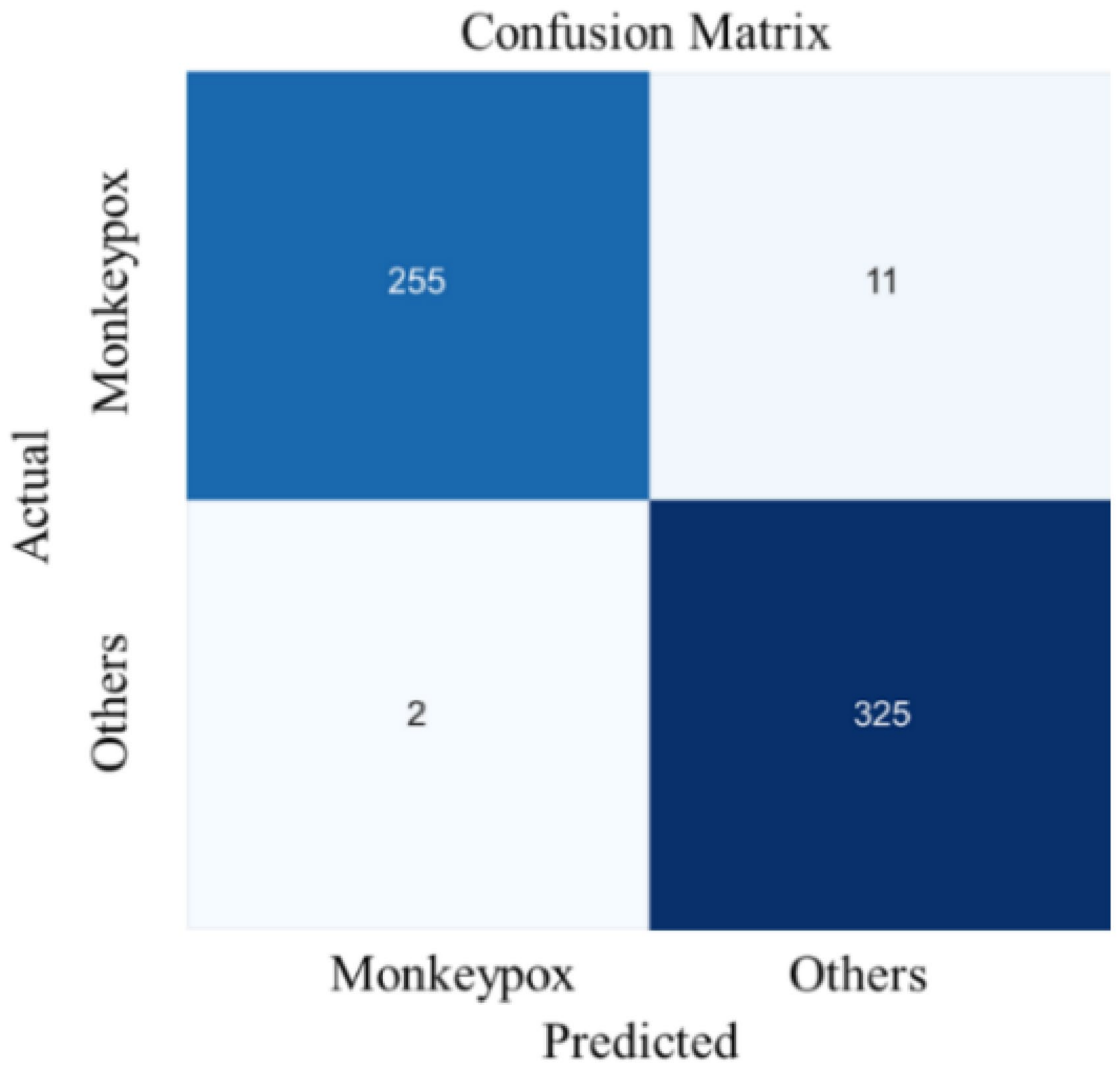
Confusion matrix for the test set.

From the perspective of complexity and inference efficiency, this study uses the EfficientNet network as the backbone of the model to extract fine-grained semantic features. The training process results of the presented model in Figure 6, which illustrates the trends in loss value and accuracy for both the training and validation sets over the course of model training. Figure 7 displays the confusion matrix for the test set.

As shown in Figure 6, the loss values for both the training and validation sets gradually decrease as the number of iterations increases during the training. After reaching a relatively flat phase, the loss approaches zero, with the minimum validation loss occurring at epoch = 20. Additionally, the figure indicates that the accuracy for both the training and validation sets steadily increases, stabilizing around 0.96 in the later stages. This suggests that the model training is nearing completion and that the model’s performance is becoming stable, indicating a convergence toward an optimal direction.

Figure 7 presents the prediction statistics for two types of samples in the test set. The result indicates that 13 samples (*FP*+*FN*) in the test set were misclassified, while 580 samples (*TP*+*TN*) were correctly classified. Additionally, 11 of the 13 misclassified samples originate from the true Mpox data, while only 2 are from the non-Mpox data. This suggests that the model is more likely to misclassify Mpox cases as non-Mpox, rather than the reverse. From the recall perspective, the performance may be more reliable than the diagnostic results of medical staff who are encountering Mpox for the first time. Given their limited knowledge of Mpox and lack of experience or awareness of the risks, these medical professionals may be less likely to suspect Mpox and may incorrectly diagnose it as another condition. Therefore, the deep learning model can be considered a valuable diagnostic aid for medical staff, providing crucial predictive insights that can enhance diagnostic accuracy.

**Figure 8:**
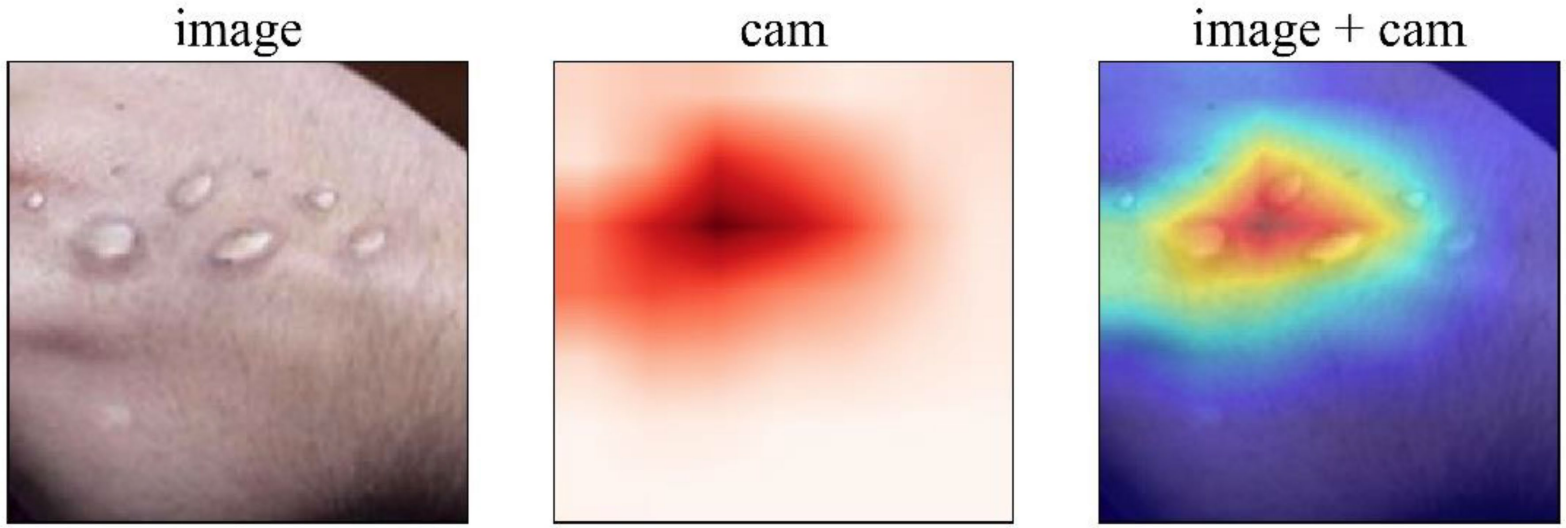
The visualization of the GRAD-CAM results on a case.

To enhance the interpretability of the model, the GRAD-CAM method is used here to extract the regions of interest that the model focuses on in the input image, as shown in Figure 8. GRAD-CAM generates class activation maps (CAMs) by computing the gradient of the target class with respect to the feature maps of the final convolutional layer, which visualizes the rationale behind the model’s decision-making process. In the figure, the original image depicts the symptoms of Mpox on the skin. The CAM image (cam) presents the model’s attention to different areas of the image in the form of a heatmap, where the redder areas indicate higher attention from the model. The overlaid image (image+cam) clearly highlights the regions of the Mpox lesions that the model predominantly focuses on. From the visualization, it can be observed that the model accurately concentrates on key features of Mpox. This demonstrates that the proposed model not only achieves high recognition accuracy but also effectively utilizes the pathological characteristics of Mpox for decision-making, further validating the model’s effectiveness. Moreover, this visualization method provides clinical practitioners with an intuitive reference to understand the model’s decision-making process, facilitating its adoption and increasing trust in real-world medical scenarios.

#### 3.3.2 Ablation experiments

In addition to stability and efficiency, strong scalability is another notable character of EfficientNet. In general, model performance is influenced by multiple parameters simultaneously, rather than by just one. The optimal approach is to consider the depth, width, and image resolution of the network concurrently when tuning neural network parameters. The EfficientNet model was developed with this principle in mind.

To this end, using the basic EfficientNet network introduced in Section 2.2 as the baseline model (EfficientNetB0), this study further explores the model performance and time efficiency when using EfficientNet extended models and other neural networks as the backbone. Four extended versions of EfficientNetB0 (EfficientNetB1 to EfficientNetB4) are trained and tested, along with VGG (VGG16 and VGG19) and ResNet models (ResNet50, ResNet101, and ResNet152). All results will be compared with the baseline model. Table 3 presents the details of the EfficientNet series, showing that the key parameters are adjusted accordingly when the baseline model is expanded. The last column in Table 3 shows the number of parameters for different extended models. It reveals that, as the model gradually expands, the number of parameters increases rapidly, meaning that the model complexity correspondingly rises.

**Table 3:** Key parameters of different extended versions of the EfficientNet model.

Items	Image pixel	Model width	Network depth	Drop rate	Total number of parameters (M)
EfficientNetB0	224×224	1.0	1.0	0.2	4.14
EfficientNetB1	240×240	1.0	1.1	0.2	6.53
EfficientNetB2	260×260	1.1	1.2	0.3	7.69
EfficientNetB3	300×300	1.2	1.4	0.3	10.58
EfficientNetB4	380×380	1.4	1.8	0.4	17.18

**Figure 9:**
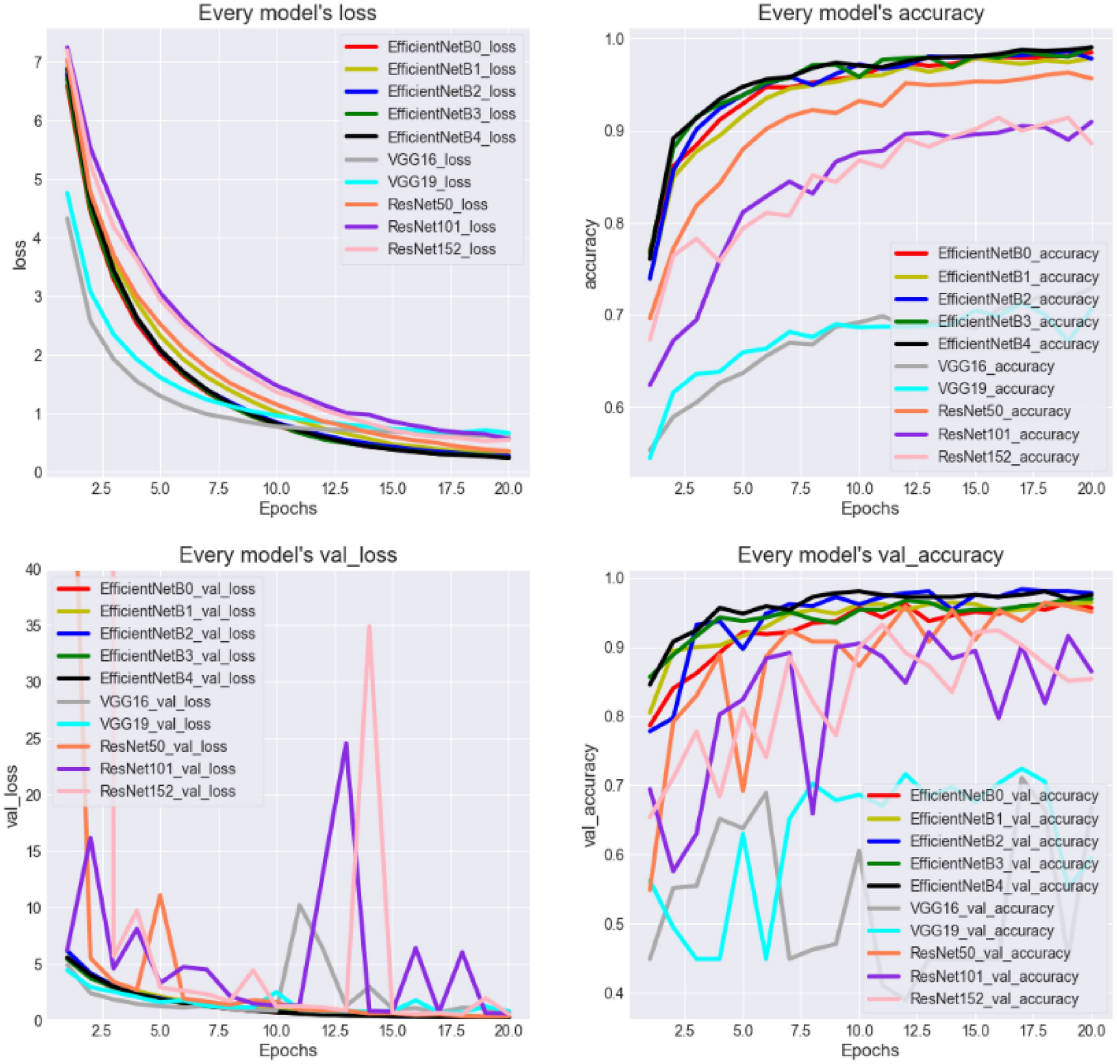
Change curve of loss value and accuracy rate of different models.

Figure 9 provides an intuitive depiction of the loss and accuracy curves for different models over time. From the figure, it is evident that the EfficientNet models perform the best, with the *accuracy* reaching approximately 96% on the validation set. This suggests that most suspected samples can be accurately identified. The ResNet models show slightly less effective performance, while the VGG models perform the worst. Furthermore, it is observed that EfficientNetB2 achieves a relatively higher accuracy, but the *accuracy* fluctuates over time, making it difficult to conclusively assert that EfficientNetB2 is the optimal model. Notably, the most complex models do not consistently outperform the simpler models, such as the performance of ResNet50 surpasses that of ResNet152. It suggests that there is insufficient evidence to support the hypothesis that a more complex model structure necessarily leads to better performance in this task.

**Figure 10:**
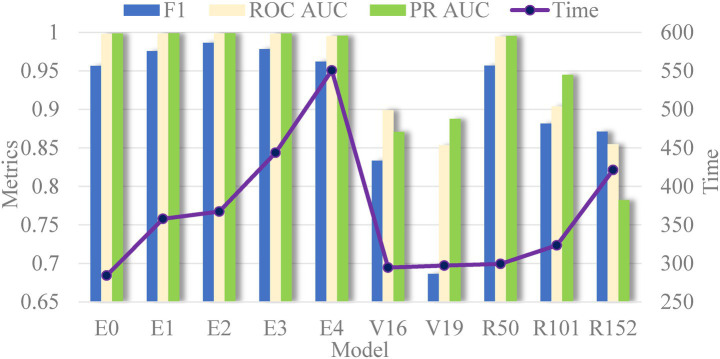
Comparison of all evaluation index values in the test set in different models (E, V and R represent the EfficientNet, VGG, and ResNet networks, respectively).

Next, Figure 10 presents the evaluation metrics of various models on the test set. Interestingly, the core evaluation metrics of the EfficientNet series networks show a pattern of initially rising and then gradually declining, with the model using EfficientNetB2 as the backbone performing the best. The reason for this trend is that, as increasing the model complexity, the model’s ability to capture semantic information strengthens, leading to improved performance. However, when the network version expands to higher levels, the excessive model parameters cause slight overfitting, resulting in a slight decline in performance. This overfitting phenomenon is particularly noticeable in the VGG and ResNet networks. Additionally, it is evident that the model’s time complexity is closely related to the number of parameters, meaning that models with less parameters are preferred in clinical scenarios requiring rapid response. Thus, selecting networks should take into account the requirements of real-world scenarios.

#### 3.3.3 Comparative analysis

To verify the superior recognition performance and lower model’s complexity of our method, we selected several advanced models for comparison. These include: (1) VGG16, which is composed of multiple stacked convolutional layers; (2) ResNet50, which not only consists of multiple convolutional layers but also uses residual networks to prevent overfitting; (3) Xception, which uses multi-scale convolutions to extract local information of different granularities; (4) MobileNet, which achieves model lightweighting using depthwise separable convolutions; (5) TMS, which combines Transformer and SVM; and (6) MxSLDNet, which uses digital twin technology for Mpox recognition.

Table 4 presents the performance and parameter count of different models. The results show that the model proposed in this study, which uses EfficientNet as the backbone, has the fewest parameters, only 4.14 M, and achieves the optimal value in most evaluation metrics due to its internal MBConv module. It indicates that the proposed model significantly reduces the parameter’s amount and computational load, making it more efficient in small-sample training and effectively preventing overfitting.

**Table 4:** The performances and the number of parameters of various models.

Models	Precision (%)	Recall (%)	F1 (%)	ROC AUC	PR AUC	Total number of parameters (M)
VGG16	83.61	83.29	83.33	0.899	0.871	14.16
ResNet50	95.7	95.69	95.68	0.995	0.996	22.95
Xception	70.48	73.12	73.12	–	–	21.85
MobileNet	90.24	90.25	90.26	–	–	4.20
TMS	95.51	95.45	95.46	0.980	–	~9.53
MxSLDNet	**96.00**	95.00	95.00	–	–	–
Our model	95.92	**95.69**	**95.80**	**0.998**	**0.999**	**4.14**

The optimal value of every indicator is highlight in bold.

Further analysis reveals the following results: (1) VGG16 and Xception only use a simple stacked structure with multiple convolutional layers, leading to a large parameter count but low feature extraction efficiency. (2) ResNet50 introduces residual connections to address the vanishing gradient problem in deep networks, enabling the extraction of more complex features, but the parameter scale increases drastically as the network depth increases. (3) MobileNet primarily achieves lightweighting by using depthwise separable convolutions, but it sacrifices some feature extraction completeness and accuracy, reducing model performance. (4) TMS may have advantages in handling large-scale data, but its complex structure leads to a larger computing amount and insufficient performance stability in small-sample Mpox recognition tasks. (5) MxSLDNet may has certain advantages in data generation, but it is less effective than our model in optimizing the feature extraction efficiency.

In a word, the performance differences between models are significant, and the complexities also vary greatly. This indicates that not all deep learning models are suitable for the small-sample data scenarios of Mpox recognition.

#### 3.3.4 Robustness analysis

To evaluate the robustness of the model proposed, a 5-fold cross-validation approach is employed. In 5-fold cross-validation, the dataset is divided into 5 equal parts, with 4 parts selected as the training set to train the models and the remaining part used for testing. This process is repeated 5 times, and the performance metrics are then averaged. To assess whether the performance differences are statistically significant, the *t-test* method is applied. If the *p*-value is greater than 0.05, it indicates no significant performance difference, suggesting the model exhibits good robustness. Conversely, if the *p*-value is less than 0.05, it implies that the model lacks robustness.

Given the requirement for rapid response, EfficientNet-B0 is selected as the backbone module of this model. The results are shown in Table 5. According to Table 5, the *precision*, *recall*, and *F1* score consistently range from 94.73 to 97.4%. The areas under the ROC curve and PR curve both remain above 0.99, indicating excellent classification performance. Additionally, the model’s time complexity stabilizes around 252 s. Notably, the *p*-values for all metrics exceed 0.05, suggesting that the results are statistically significant. These findings collectively demonstrate that the proposed model passes the robustness test following cross-validation.

**Table 5:** The outcomes of EfficientNetB0 in every cross validation.

Item	Precision (%)	Recall (%)	F1 (%)	ROC-AUC	PR-AUC	Time Complexity
1	95.91	95.78	95.77	0.991	0.991	251.56
2	96.56	96.46	96.45	0.992	0.992	251.62
3	95.16	94.77	94.73	0.993	0.993	252.49
4	97.35	97.3	97.3	0.997	0.998	252.17
5	97.4	97.3	97.29	0.999	0.999	251.91
mean	96.48	96.32	96.31	0.994	0.995	251.95
*p*	>0.05	>0.05	>0.05	>0.05	>0.05	>0.05

### 3.4 Conclusion

In the context of Mpox recognition, to meet the efficiency requirement in clinical practice, this study proposes a lightweight model utilizing transfer learning techniques. Specifically, on the one hand, the model employs the EfficientNet network as the backbone, where the internal MBConv module combines depthwise separable convolutions and the SE attention mechanism, effectively reducing the model’s parameter scale and computational complexity. This lightweight model better aligns with the stringent efficiency requirement in clinical practice. On the other hand, the model leverages transfer learning to transfer parameters from the pre-trained EfficientNet network on the large-scale ImageNet dataset to this model. This approach effectively utilizes the existing feature extraction capabilities of the network, enhancing the model’s performance and stability in small-sample datasets.

This study has verified the effectiveness and stability of the proposed method through several experiments. Based on the analysis, the following conclusions can be drawn:

1. Compared with existing advanced models, the model in this study, using EfficientNet as the backbone, achieved the optimal values in most metrics with the smallest parameter scale. Specifically, the *precision*, *recall*, *F1* score, *ROC-AUC*, and *PR-AUC* values were 95.92, 95.69, 95.80%, 0.998, and 0.999, respectively. These results demonstrate the efficiency and effectiveness of the proposed model in this task.
2. To minimize model complexity, EfficientNetB0 is selected as the base model’s backbone, and the 5-fold cross-validation is used to validated its robustness. The average values for model *precision*, *recall*, *F1* score, *ROC-AUC*, and *PR-AUC* are 96.48, 96.32, 96.31%, 0.994, and 0.995, respectively, with *p* > 0.05. This indicates that there are no significant differences in the model’s performance metrics, confirming that it passes the robustness test.
3. The VGG and ResNet models are compared to the baseline models. The best *F1* scores for the extended models of EfficientNet, ResNet, and VGG are 98.65, 95.68, and 83.33%, respectively. This shows that VGG significantly underperforms compared to both EfficientNet and ResNet, with EfficientNet outperforming ResNet. It indicates that not all deep learning models are suitable for the Mpox identification task.
4. In specific practical scenarios, it is necessary to choose the appropriate network as the model’s backbone based on the requirements. For example, when faster inference speed is needed, the lightweight EfficientNetB0 network can be used as the backbone, which slightly reduces performance but significantly improves training speed. If higher accuracy is required, the EfficientNetB2 network can be used.

## 4 Discussion

The speed and scope of the current Mpox outbreak are significantly higher than previous instances, making it a serious international health event. As a result, the WHO has urgently classified it as a Public Health Emergency of International Concern (PHEIC), calling for increased attention from all countries and regions. A distinctive feature of this outbreak is its emergence in areas where Mpox has never been reported before. For many healthcare workers, this marks the first encounter with this unfamiliar disease, and they may misdiagnose Mpox as other conditions due to a lack of sufficient clinical experience or heightened risk awareness. Consequently, there is a pressing need to provide advanced diagnostic support technology for medical personnel.

To address this need, this study proposes a deep learning model to identify Mpox among a range of suspected skin diseases. The model leverages transfer learning technology to identify Mpox among a range of suspected skin diseases. By providing diagnostic assistance, this method aims to support frontline healthcare workers, enabling them to focus on specific patients and cases, thereby minimizing the potential for Mpox transmission within the population.

Compared with these advanced models, the evaluation results indicate that the model with EfficientNetB0 performs well in the task, as well as its extended models show great generalizability and versatility. However, not all deep learning models are equally suitable for this task, as evidenced by the poor performance of the VGG model. Moreover, although TMS utilizes the Transformer to achieve model lightweighting and achieves performance similar to that of our model, its internal use of a full self-attention mechanism increases computational complexity, which imposes high hardware requirements.

In a word, this study found that deep learning models are capable of effectively performing this task and can provide high-performance diagnostic support tools for frontline medical staff. It is important to note that, during the application phase, decision-makers must select models based on their specific requirements. If rapid model training and result generation are prioritized, EfficientNetB0 should be preferred, due to its relatively simple architecture, fewer parameters, and faster convergence. On the other hand, if more stable model performance is required, EfficientNetB2 may be a better choice, based on the current results.

It is important to note that deep learning technology serves as an auxiliary tool to help inexperienced medical staff reduce the risk of misdiagnosis. However, to fundamentally control the spread of Mpox, managers must focus on the following aspects: encouraging vaccination, enhancing public awareness and education (Kotwal et al., 2024), strictly managing virus samples, regulating the trade of animals to prevent the spread of Mpox, and discouraging the consumption of wild animals or unquarantined meats. Only through the full implementation of these measures can the spread and infection of the virus be effectively prevented.

A limitation of this study is the small scale of the available sample data, which restricts the range of information it covers. Notably, it lacks data from diverse skin color populations, which could pose a risk of misjudgments when the model encounters feature it has not previously learned. Therefore, a key direction for future research is to expand the dataset. This could involve collecting more data on factors such as skin color, age groups, and non-Mpox diseases from a broader range of platforms. Additionally, in terms of model development, on one hand, exploring possible pathways to reduce the computational complexity of Transformer to enhance its usage efficiency. On the other hand, enhancing the interpretability of the model’s predictions, which is a hot-topic of great concern to clinical practitioners. Moreover, exploring the use of federated learning technology could be beneficial. This technology would allow the use of Mpox diagnostic data from other medical institutions while ensuring patient privacy.

## Data Availability

The original contributions presented in the study are included in the article/supplementary material, further inquiries can be directed to the corresponding author.

## Footnotes

^1^https://news.un.org/zh/story/2022/07/1106492, 2022-07-23/2022-09-21.

^2^https://www.sohu.com/a/574701774_162758, 2022-08-06/2022-09-23.

^3^http://usa.people.com.cn/n1/2022/0926/c241376-32534032.html, 2022-08-06/ 2022-09-24.

## References

[ref1] Abdelrahim E. M. Hashim H. Atlam E. S. Osman R. A. Gad I. (2024). Tms: ensemble deep learning model for accurate classification of monkeypox lesions based on transformer models with svm. Diagnostics (Basel) 14. doi: 10.3390/diagnostics14232638, PMID: 39682546 PMC11639930

[ref2] Aboulmira A. Hrimech H. Lachgar M. Hanine M. Garcia C. O. Mezquita G. M.. (2025). Hybrid model with wavelet decomposition and EfficientNet for accurate skin cancer classification. J. Cancer 16, 506–520. doi: 10.7150/jca.101574, PMID: 39744476 PMC11685683

[ref3] Al-Gaashani M. S. A. M. Xu W. Obsie E. Y. (2025). MobileNetV2-based deep learning architecture with progressive transfer learning for accurate monkeypox detection. Appl. Soft Comput. 169:112553. doi: 10.1016/j.asoc.2024.112553

[ref4] Almars A. M. (2025). Deepgenmon: a novel framework for monkeypox classification integrating lightweight attention-based deep learning and a genetic algorithm. Diagnostics (Basel) 15:130. doi: 10.3390/diagnostics15020130, PMID: 39857013 PMC11763561

[ref5] Alruwaili M. Mohamed M. (2025). An integrated deep learning model with EfficientNet and ResNet for accurate multi-class skin disease classification. Diagnostics 15:551. doi: 10.3390/diagnostics15050551, PMID: 40075797 PMC11898587

[ref6] Andrieu J. Mège J.-L. Mezouar S. (2025). Monkeypox virus and pregnancy. J. Med. Virol. 97:e70337. doi: 10.1002/jmv.70337, PMID: 40223710 PMC11995370

[ref7] Arora L. Singh S. K. Kumar S. Gupta H. Alhalabi W. Arya V.. (2024). Ensemble deep learning and EfficientNet for accurate diagnosis of diabetic retinopathy. Sci. Rep. 14:30554. doi: 10.1038/s41598-024-81132-4, PMID: 39695310 PMC11655640

[ref8] Bala D. Hossain M. S. Hossain M. A. Abdullah M. I. Rahman M. M. Manavalan B.. (2023). MonkeyNet: a robust deep convolutional neural network for monkeypox disease detection and classification. Neural Netw. 161, 757–775. doi: 10.1016/j.neunet.2023.02.022, PMID: 36848828 PMC9943560

[ref9] Cap Q. H. Uga H. Kagiwada S. Iyatomi H. (2022). Leafgan: an effective data augmentation method for practical plant disease diagnosis. IEEE Trans. Autom. Sci. Eng. 19, 1258–1267. doi: 10.1109/TASE.2020.3041499

[ref10] De Vries I. R. Melaet R. Huijben I. A. M. Van Laar J. O. E. H. Kok R. D. Oei S. G.. (2025). Conditional contrastive predictive coding for assessment of fetal health from the cardiotocogram. IEEE J. Biomed. Health Inform. 29, 3377–3386. doi: 10.1109/JBHI.2025.3530610, PMID: 40030899

[ref11] Di Giulio D. B. Eckburg P. B. (2004). Human monkeypox: An emerging zoonosis. Lancet Infect. Dis. 4, 15–25. doi: 10.1016/S1473-3099(03)00856-9, PMID: 14720564 PMC9628772

[ref12] Dong Z. M. Hu Q. Zhang Z. Y. Zhao J. J. (2024). On the effectiveness of graph data augmentation for source code learning. Knowl.-Based Syst. 285:328. doi: 10.1016/j.knosys.2023.111328

[ref13] Elhadidy M. S. Elgohr A. T. El-Geneedy M. Akram S. Kasem H. M. (2025). Comparative analysis for accurate multi-classification of brain tumor based on significant deep learning models. Comput. Biol. Med. 188:109872. doi: 10.1016/j.compbiomed.2025.109872, PMID: 39970824

[ref14] Guarner J. (2022). Monkeypox in 2022 a new outbreak of an old disease. Am. J. Clin. Pathol. 158, 160–161. doi: 10.1093/ajcp/aqac091 35751634

[ref15] Haque M. A. Halder A. S. Hossain M. J. Islam M. R. (2024). Prediction of potential public health risk of the recent multicountry monkeypox outbreak: an update after the end declaration of global public health emergency. Health Sci. Rep. 7:2136. doi: 10.1002/hsr2.2136, PMID: 38817885 PMC11136639

[ref16] Haque R. Sultana A. Haque P. (2023). *Ensemble of fine-tuned deep learning models for monkeypox detection: A comparative study*. 2023 4th international conference for emerging technology (Incet).

[ref17] Jahan S. Ge Y. F. Kabir E. Wang K. (2025). Analysis and multi-objective protection of public medical datasets from privacy and utility perspectives. Data Sci. Eng. 2025:283. doi: 10.1007/s41019-025-00283-0

[ref18] Jaradat A. S. Al Mamlook R. E. Almakayeel N. Alharbe N. Almuflih A. S. Nasayreh A.. (2023). Automated monkeypox skin lesion detection using deep learning and transfer learning techniques. Int. J. Environ. Res. Public Health 20:422. doi: 10.3390/ijerph20054422, PMID: 36901430 PMC10001976

[ref19] Kameli N. Algaissi A. Taha M. M. E. Alamer E. Alhazmi A. Hakami W.. (2025). Monkeypox global research: a comprehensive analysis from emergence to present (1961-2023) for innovative prevention and control approaches. J. Infect. Public Health 18:102593. doi: 10.1016/j.jiph.2024.102593, PMID: 39608220

[ref20] Kotwal J. Kashyap R. Shafi P. M. Kimbahune V. (2024). Enhanced leaf disease detection: Unet for segmentation and optimized EfficientNet for disease classification. Softw. Impacts 22:100701. doi: 10.1016/j.simpa.2024.100701

[ref21] Kumari L. V. R. Jagruti K. Chandra G. R. Reddy M. S. Bhadramma B. (2024). Transfer learning based EfficientNet for knee osteoarthritis classification. Traitement Sig 41, 989–997. doi: 10.18280/ts.410239

[ref22] Lee H. Cho S. Song J. Kim H. Shin Y. (2025). An enhanced approach using Ags network for skin cancer classification. Sensors 25:394. doi: 10.3390/s25020394, PMID: 39860766 PMC11769443

[ref23] Lin G. Jiang J. Z. Bai J. Su Y. W. Su Z. H. Liu H. S. (2025). Frontiers and developments of data augmentation for image: from unlearnable to learnable. Inf. Fusion 114:102660. doi: 10.1016/j.inffus.2024.102660

[ref24] Maqsood S. Damaševičius R. Shahid S. Forkert N. D. (2024). Mox-net: multi-stage deep hybrid feature fusion and selection framework for monkeypox classification. Expert Syst. Appl. 255:124584. doi: 10.1016/j.eswa.2024.124584

[ref25] Mehmood A. Gulzar Y. Ilyas Q. M. Jabbari A. Ahmad M. Iqbal S. (2023). Sbxception: a shallower and broader Xception architecture for efficient classification of skin lesions. Cancers (Basel) 15:604. doi: 10.3390/cancers15143604, PMID: 37509267 PMC10377736

[ref26] Pramanik R. Banerjee B. Efimenko G. Kaplun D. Sarkar R. (2023). Monkeypox detection from skin lesion images using an amalgamation of Cnn models aided with Beta function-based normalization scheme. PLoS One 18:e0281815. doi: 10.1371/journal.pone.0281815, PMID: 37027356 PMC10081766

[ref27] Raha A. D. Gain M. Debnath R. Adhikary A. Qiao Y. Hassan M. M.. (2024). Attention to monkeypox: an interpretable monkeypox detection technique using attention mechanism. IEEE Access 12, 51942–51965. doi: 10.1109/ACCESS.2024.3385099

[ref28] Ravi V. (2022). Attention cost-sensitive deep learning-based approach for skin cancer detection and classification. Cancer 14:872. doi: 10.3390/cancers14235872, PMID: 36497355 PMC9735681

[ref29] Ribas L. C. Casaca W. Fares R. T. (2025). Conditional generative adversarial networks and deep learning data augmentation: a multi-perspective data-driven survey across multiple application fields and classification architectures. AI 6:32. doi: 10.3390/ai6020032

[ref30] Saxena S. K. Ansari S. Maurya V. K. Kumar S. Jain A. Paweska J. T.. (2023). Re-emerging human monkeypox: a major public-health debacle. J. Med. Virol. 95:e27902. doi: 10.1002/jmv.27902, PMID: 35652133

[ref31] Shi Y. Abulizi A. Wang H. Feng K. Abudukelimu N. Su Y.. (2025). Diffusion models for medical image computing: a survey. Tsinghua Sci. Technol. 30, 357–383. doi: 10.26599/TST.2024.9010047

[ref32] Sitaula C. Shahi T. B. (2022). Monkeypox virus detection using pre-trained deep learning-based approaches. J. Med. Syst. 46:78. doi: 10.1007/s10916-022-01868-2, PMID: 36201085 PMC9535233

[ref33] Tigrini A. Sbrollini A. Mengarelli A. (2025). Data processing and machine learning for assistive and rehabilitation technologies. Bioengineering 12:70. doi: 10.3390/bioengineering12010070, PMID: 39851344 PMC11762809

[ref34] Tripathi P. Pandey S. Yadav D. Joshi S. (2025). Emergence and evolution of monkeypox virus: epidemiology, pathology, clinical symptoms, preventative and treatment measures. Int. Immunopharmacol. 152:114448. doi: 10.1016/j.intimp.2025.114448, PMID: 40073815

[ref35] Vasavi G. Rani V. V. Ponnada S. Jyothi S. (2025). A hybrid EfficientNet-DbneAlexnet for brain tumor detection using MRI images. Comput. Biol. Chem. 115:108279. doi: 10.1016/j.compbiolchem.2024.108279, PMID: 39631224

[ref36] Wallau G. L. Maciel-De-Freitas R. Schmidt-Chanasit J. (2022). An unfolding monkeypox outbreak in Europe and beyond. Mil. Med. Res. 9:31. doi: 10.1186/s40779-022-00394-z, PMID: 35706050 PMC9198408

[ref37] Wang W. J. Jiang X. Y. Yuan H. Chen J. Y. Wang X. T. Huang Z. C. (2024). Research on algorithm for authenticating the authenticity of calligraphy works based on improved EfficientNet network. Appl. Sci.-Basel 14:295. doi: 10.3390/app14010295

[ref38] Wang J. Liu Q. Y. Xie H. T. Yang Z. G. Zhou H. F. (2021). Boosted EfficientNet: detection of lymph node metastases in breast cancer using convolutional neural networks. Cancer 13:661. doi: 10.3390/cancers13040661, PMID: 33562232 PMC7915222

[ref39] Wilder-Smith A. Osman S. (2020). Public health emergencies of international concern: a historic overview. J. Travel Med. 27:227. doi: 10.1093/jtm/taaa227, PMID: 33284964 PMC7798963

[ref40] Yang S. R. Li J. Q. Zhang T. Y. Zhao J. Shen F. R. (2023). Advmask: a sparse adversarial attack-based data augmentation method for image classification. Pattern Recogn. 144:847. doi: 10.1016/j.patcog.2023.109847

[ref41] Zhang C. L. Bao N. Sun H. Li H. Li J. Qian W.. (2022). A deep learning image data augmentation method for single tumor segmentation. Front. Oncol. 12:988. doi: 10.3389/fonc.2022.782988, PMID: 35237511 PMC8882602

[ref42] Zhang Y. Huang S. Huangfu L. W. Zeng D. D. (2025). Learning feature exploration and selection with handcrafted features for few-shot learning. IEEE Trans. Syst. Man Cyber. Syst. 55, 2599–2610. doi: 10.1109/TSMC.2024.3524390

